# Facilitators, barriers and strategies for health-system guidance implementation: a critical interpretive synthesis protocol

**DOI:** 10.1186/s12961-022-00908-0

**Published:** 2022-09-29

**Authors:** Qi Wang, Ying Zhu, Shitong Xie, Mohammad Golam Kibria, Qiangqiang Guo, Ahmed Atef Belal, Yanfei Li, Jingyi Zhang, Yaolong Chen, Holger J. Schünemann, Michael G. Wilson, Kehu Yang, John N. Lavis

**Affiliations:** 1 Health Policy Ph.D. Program, McMaster University, Hamilton, ON Canada; 2 Evidence Based Social Science Research Center, School of Public Health, Lanzhou University, Lanzhou, China; 3 McMaster Health Forum, McMaster University, Hamilton, ON Canada; 4 Department of Health Research Methods, Evidence and Impact, Faculty of Health Sciences, McMaster University, Hamilton, ON Canada; 5 School of Pharmaceutical Science and Technology, Tianjin University, Tianjin, China; 6 Canadian Red Cross, Ottawa, ON Canada; 7 Evidence-Based Medicine Center, School of Basic Medical Sciences, Lanzhou University, Lanzhou, China; 8 Key Laboratory of Evidence Based Medicine and Knowledge Translation of Gansu Province, Lanzhou, China; 9 Center for Clinical Epidemiology and Methodology (CCEM), Guangdong Second Provincial General Hospital, Guangzhou, China; 10 Research Unit of Evidence-Based Evaluation and Guidelines, Chinese Academy of Medical Sciences (2021RU017), School of Basic Medical Sciences, Lanzhou University, Lanzhou, China; 11 WHO Collaborating Centre for Guideline Implementation and Knowledge Translation, Lanzhou University, Lanzhou, China; 12 Department of Medicine, McMaster University, Hamilton, ON Canada; 13 Michael G DeGroote Cochrane Canada and GRADE Centres, Hamilton, ON Canada; 14 Centre for Health Economics and Policy Analysis, McMaster University, Hamilton, ON Canada; 15 Africa Centre for Evidence, University of Johannesburg, Johannesburg, South Africa

**Keywords:** Health system guidance, Implementation, Facilitators, Barriers, Strategies, Theoretical framework

## Abstract

**Background:**

As systematically developed statements regarding possible courses of action, health system guidance (HSG) can assist with making decisions about addressing problems or achieving goals in health systems. However, there are conceptual and methodological challenges in HSG implementation due to the complexity of health-system policy-making, the diversity of available evidence and vast differences in contexts. To address these gaps, we aim to develop a theoretical framework for supporting HSG implementation as part of a broader effort to promote evidence-informed policy-making in health systems.

**Methods:**

To develop a theoretical framework about facilitators, barriers and strategies for HSG implementation, we will apply a critical interpretive synthesis (CIS) approach to synthesize the findings from a range of relevant literature. We will search 11 electronic databases and seven organizational websites to identify relevant published and grey literature. We will check the references of included studies and contact experts to identify additional eligible papers. Finally, we will conduct purposively sampling of the literature to fill any identified conceptual gaps. We will use relevance and five quality criteria to assess included papers. A standardized form will be developed for extracting information. We will use an interpretive analytic approach to synthesize the findings, including a constant comparative method throughout the analysis. Two independent reviewers will conduct the literature screening and relevance assessment, and disagreements will be resolved through discussion. The principal investigator will conduct data extraction and synthesis, and a second reviewer will check the sample of extracted data for consistency and accuracy.

**Discussion:**

A new theoretical framework about facilitators, barriers and strategies for HSG implementation will be developed using a CIS approach. The HSG implementation framework could be widely used for supporting the implementation of HSG covering varied topics and in different contexts (including low-, middle- and high-income countries). In later work, we will develop a tool for supporting HSG implementation based on the theoretical framework.

*Registration* PROSPERO CRD42020214072. Date of Registration: 14 December 2020.

**Supplementary Information:**

The online version contains supplementary material available at 10.1186/s12961-022-00908-0.

## Background

Health systems can be understood and conceptualized from numerous perspectives. According to the *World Health Report 2000*, WHO defines health systems as “all the activities whose primary purpose is to promote, restore or maintain health” [1]. To better conduct research in health systems, based on the systematic analysis of current health system frameworks, a WHO-commissioned panel defines health systems as “the governance, financial and delivery arrangements for health care and public health services, implementation considerations for reforming or strengthening these arrangements, and broader economic, legal, political and social contexts in which these arrangements are negotiated and operate” [2]. However, soundly defining health systems is just one step among many towards strengthening such systems such that they can reliably achieve the healthcare “quadruple aim” [3] or the health targets of the Sustainable Development Goals (SDGs). According to *World health statistics 2019*, there have been improvements in health-related SDG indicators, such as increases in global and healthy life expectancy and decreases in neonatal deaths. However, disparities and inequities in health outcomes within and among countries remain, with substantial room for improvement [4].

Policy-makers, health providers, researchers and other stakeholders need to work together to ensure that health-system arrangements are optimized to achieve the right mix of safe, effective and cost-effective health interventions to those who need them. However, multiple studies show that weaknesses in these system arrangements—a lack of trained health workers, a lack of a robust infrastructure, an unreliable supply of medicines and technologies, and inadequate funding—limit the reach and impacts of such interventions [5, 6]. Therefore, it is crucial that action be taken to strengthen health systems in general and to implement a “learning health system” specifically [7].

Just as physicians modify their practice based on clinical guidelines, policy-makers can develop or adjust policies based on health-system guidance (HSG). Such guidance generally contains proposed options and supporting evidence. HSG has been defined as “systematically developed statements produced at global or national levels to assist decisions about appropriate options for addressing a health system challenge in a range of settings and to assist with the implementation of these options and their monitoring and evaluation” [8]. When HSG is produced at the global level, it is generally used to support decisions or policies of national (or subnational) governments and international organizations that would contribute to strengthening health systems [8, 11, 12].

For example, WHO produces HSG to be used by its Member States as well as its own staff in their work with Member States, as it has done on topics such as increasing access to and retention of health workers in remote and rural areas in 2010 [9] and supporting the optimization of community health worker roles in 2018 [10]. Bosch-Capblanch et al. have articulated the rationale for and identified the challenges in HSG [8]. WHO commissioned an expert panel to produce a handbook to assist with HSG development [13]. Others have described the complexity of health systems (the individual components and their multidirectional interactions), the diversity of evidence related to health systems, and the highly context-sensitive and multifactorial policy-making process [13, 14] that contribute to conceptual, methodological and practical challenges with HSG [8, 11–13]. Still others have proposed principles and strategies for better supporting HSG adaptation and implementation [11], and have begun to develop frameworks, approaches and tools to ensure quality in development [15] and contextualization [16] processes.

As many of these articles and reports allude to, the implementation context for HSG is more multilayered and complicated than that for clinical practice guidelines (CPG) and public health guidance (PHG). Although there are some well-developed implementation strategies, frameworks and tools for health guidelines [17–23], the vast majority focus on CPG implementation, not HSG implementation [13].

Therefore, there is a need for a comprehensive, systematic, well-organized theoretical framework and practical tool to support the implementation of global HSG at the national or subnational level. The first step in the research programme, and the focus of this study, is to conduct a knowledge synthesis of the published studies and grey literature to identify facilitators, barriers and strategies—at the individual, organizational, community and system levels—related to HSG implementation. The synthesis results will provide the research community with a comprehensive HSG implementation theoretical framework.

## Methods/design

### Objective

Our overarching objective is to develop a theoretical framework concerning the facilitators of, barriers to and strategies for HSG implementation at different levels based on the following two compass questions:

1. What factors—at the individual, organizational, community and system levels—facilitate or hinder HSG implementation processes and outcomes?
2. What strategies—at the individual, organizational, community and system levels—can leverage facilitators of and address barriers to HSG implementation processes and outcomes?

### Study design

We will use a critical interpretive synthesis (CIS) approach to develop a theoretical framework. As a knowledge synthesis approach, CIS can address some of the limitations of traditional systematic review methodology by combining the qualitative inquiry technique [24]. The CIS approach is used to develop concepts and theories through an inductive, interpretive and iterative process. Instead of data aggregation, the output of a CIS will be the theory generated from included studies drawn from a large, diverse and complex body of literature. Both the process and output of the CIS are conceptual [24–26]. Since Dixon-Woods et al. introduced the CIS method with an example of access to healthcare by vulnerable populations [24], the CIS approach has been applied to generate or revise theories or theoretical frameworks, especially in fields with a large and complex body of literature [15, 27, 28]. For example, Ako-Arrey and colleagues used a CIS approach to identify and organize 30 concepts related to HSG appraisal [15].

A CIS approach is particularly appropriate for our study for the following reasons. First, CIS is suitable for analysing and synthesizing findings from diverse and complex types of literature with varied methodologies (such as quantitative empirical studies, qualitative empirical studies, conceptual or theoretical papers) [24, 25]. The literature related to HSG is highly heterogeneous, with diverse study designs. Also, as a nascent domain, HSG is often categorized as part of CPG or PHG or “general” health guidelines [15]. Second, a CIS is used to develop concepts and theories based on a detailed inspection of literature, an iterative and flexible process of inquiry and an inductive interpretation [24].

Since there is no widely accepted reporting guideline for the CIS protocol, we will describe our CIS protocol following the Preferred Reporting Items for Systematic Review and Meta-Analysis Protocols (PRISMA-P) 2015 statement [29] (Additional file 1), CIS methodology paper [24] and available examples [15, 27, 28, 30].

### Five steps

#### Identifying potentially relevant articles

We will conduct the literature search in phases while guided by the two compass questions. The search strategy was developed through multiple consultations with librarians at McMaster University. The search terms included “health system”, “guidance”, “recommendation”, “guideline” and “implementation” and their synonyms and varieties (see Additional file 2 for the detailed search strategies for each database).

We will search 11 electronic databases: Chinese Biomedical Literature Database (CBM), CNKI (China National Knowledge Infrastructure), Cochrane Library, Cumulative Index to Nursing and Allied Health Literature (CINAHL), Embase, Google Scholar, HealthSTAR, Health Systems Evidence (HSE), PubMed, Wanfang Data and Web of Science. Also, we will search the following websites containing grey literature: BIGG (Base internacional de guias GRADE [Grading of Recommendations Assessment, Development and Evaluation]), Guidelines International Network (GIN), Health Systems Global, National Institute for Health and Care Excellence (NICE), National Institutes of Health (NIH), Scottish Intercollegiate Guidelines Network (SIGN) and WHO. We will check the references of included studies and contact relevant experts (including first/corresponding authors of our included studies and those with expertise in HSG and/or implementation science) to identify additional potentially eligible papers. Finally, we will conduct purposive searches to identify literature that fills any identified conceptual gaps [27].

#### Selecting potentially eligible articles

All reviewers will conduct pilot screening to reach consensus on inclusion and exclusion criteria. If necessary, the inclusion and exclusion criteria could be changed based on pilot screening results through discussion. All paired reviewers will independently screen titles and abstracts and full texts of all identified literature against our criteria to classify each article as directly relevant, indirectly relevant or excluded literature. We will resolve disagreements through discussion. The direct and indirect literature will be included in our sample pool of literature, from which we will select our purposive sample of relevant papers for the synthesis. The selection of our purposive sample is mainly based on their ability to offer vital conceptual insights related to HSG implementation, which will be reviewed and adjusted, if necessary, through online discussion among the research team members.

While we will use kappa statistics to calculate inter-rater reliability for measuring the agreement among reviewers, we acknowledge that a qualitative synthesis approach like CIS does not typically involve the use of a quantitative measure. We are doing so here to spur and improve reflexivity. We will accept kappa values of 0.61–0.80 as substantial agreement and 0.81–1.00 as perfect agreement for literature screening in our CIS [31]. During the pilot screening phase, a kappa result below 0.61 will indicate that the reviewers need further training and another round of pilot screening. After reaching “substantial agreement, we will start formal screening.

We will include all empirical and nonempirical articles based on their study content, namely those related to the facilitators of, barriers to and strategies to support HSG implementation at any level (individual, organizational, community or system). Given the great variety in terms that are used in the titles of HSG documents, such as “policy guidance”, “policy recommendations”, “guideline”, “guidance” and “recommendations”, we will adopt the HSG definition proposed by Bosch-Capblanch et al. (as mentioned above) [8] in our study. Specifically, all or most of the recommendations of HSG documents should focus on one or more aspects of health system arrangements, which could be coded with the HSE taxonomy of governance, financial and delivery arrangements in health systems (hereinafter referred to as the HSE framework) [32].

If the literature directly relevant to HSG implementation is scarce, we will consider including some papers relevant to the implementation of general CPG or PHG. We will categorize the former as “directly relevant” and the latter as “indirectly relevant”. The detailed eligibility criteria are shown in Table 1. Moreover, to achieve a comprehensive search result, we will not place any restrictions on the following aspects: (1) time frame, (2) context (including low-, middle- and high-income countries), (3) study design (including peer-reviewed publications, conference abstracts, theses and dissertations, editorials, comments, correspondence, etc.) and (4) language.

**Table 1:** Inclusion and exclusion criteria

Inclusion criteria (a publication with any study design in which the primary focus is):
• Implementation process (any facilitators, barriers, strategies, etc.) of HSG (generally or specifically)
• Implementation process (any facilitators, barriers, strategies, etc.) of CPG and/or PHG (only generally)
Exclusion criteria:
• Implementation of a specific clinical practice guideline or public health guidance
• Implementation of a health system intervention or programme
• Implementation of a public health intervention or programme
• Implementation of a clinical intervention or programme

#### Assessing relevant articles

Paired reviewers will independently assess the relevant articles, and will resolve disagreements through discussion or consulting a third reviewer. A CIS does not typically include a quality assessment but instead uses a relevance assessment. We will use relevance and five quality criteria to assess the included papers. First, we will use a flexible relevance boundary to comprehensively include the papers contributing to generating concepts and theory. The relevance criteria can be defined as the ability or the contribution to provide the concepts, theories or insights to answer the compass questions [24]. Then, given the complexity of potentially included papers, we will further exclude the fatally flawed empirical papers, which will be the second step of the literature assessment. The five quality criteria were modified based on the criteria developed by the National Health Service (NHS) National Electronic Library for Health for evaluating qualitative research. They are as follows: (1) the aims and objectives of the research are clearly stated; (2) the research design is clearly specified and appropriate for the aims and objectives of the research; (3) the researchers provide a clear account of the process by which their findings were reproduced; (4) the researchers display enough data to support their interpretations and conclusions; and (5) the method of analysis is appropriate and adequately explicated [24].

#### Extracting data and information

Data will be extracted by the principal investigator (QW), and the sample of extracted data will be checked by a second reviewer for consistency and accuracy. We have developed a standardized form (see Additional file 3) to extract the characteristics of each included paper, including title, publication year, author, publication form, study design, country focus and the implementation object.

We will extract key findings from each included paper by writing a brief summary and identifying the HSG implementation facilitators, barriers and strategies at four different levels. The facilitators, barriers and strategies will be identified if they are explicitly mentioned or referenced in the full text. Sometimes, strategies will be deduced based on the implications of the identified facilitators and barriers and the study team’s accumulated understanding or insights about the HSG implementation field [28]. The extracted facilitators, barriers and strategies will be further categorized based on four related frameworks [32–35].

For the individual level, we will use the second version of the Theoretical Domains Framework (TDF) and the Behaviour Change Wheel (BCW) to extract and categorize the findings [33, 34]. As the theoretical framework for identifying determinants of behaviour change, the TDF includes 14 domains, with a set of variables that can help explain what factors and strategies at the individual level facilitate or hinder HSG implementation processes [33]. The BCW framework centres on a “behaviour system” involving three essential conditions (capability, opportunity and motivation), which are positioned around nine intervention functions and seven categories of policy [34]. For the individual level, we will focus on providers and patients/citizens.

For the organizational and community levels, we will extract all implementation facilitators, barriers and strategies that exist or occur in the organizational or community setting based on the HSE framework [32]. For example, the organizational level will include all organizations that are involved in health systems, such as organizations for providing care (such as hospitals, clinics and pharmacies), organizations for providing funds (such as donor agencies) and other related international and/or nongovernmental organizations (such as WHO). We define the community as the local or district health system. Community care programmes differ among different communities and/or population groups, such as retirement homes, residential hospices, and exercise and falls-prevention programmes [36].

For the system level, we will separate two subcategories, health and political systems. For the aspect of the health system, we will use the HSE framework to extract and organize the findings at the health-system level [32]. For the aspect of the political system, we will use the framework of policy development and implementation (3I+E, institutions, interests, ideas and external factors) as a set of variables that can help explain the findings at the political level to categorize the political system findings [11, 14, 35].

#### Synthesizing and integrating findings

We will use an interpretive analytic approach to synthesize the findings from included papers, including a constant comparative method [37] throughout the analysis. When conceptually mapping the relevant papers to categorize the findings, we will use a 3 × 4 matrix to cross-link three aspects (facilitators, barriers and strategies) and four levels (individual, organizational, community and system). The conceptual mapping exercise will help categorize the literature into domains and topics of interest, identify some conceptual gaps, and further conduct purposive sampling to identify relevant literature [30]. During the process, iterative discussions among the research team will be conducted.

As noted above, we will use the TDF, BCW, HSE and 3I+E frameworks to organize the findings. The analysis, synthesis and integration processes will be iteratively conducted through constant discussion among our interdisciplinary research team members with relevant expertise and experience [28, 30]. Specifically, the analysis and synthesis process will involve (1) identifying common themes and concepts based on included papers; (2) developing theoretical constructs based on the emerging themes and concepts; (3) critiquing the emerging theoretical constructs as a whole and with our total sample of literature to identify conceptual gaps in the available evidence concerning our principal aims; (4) conducting additional purposive sampling of included papers and/or conducting additional purposive searches to fill conceptual gaps (if needed) until theoretical saturation is reached (i.e. in our study, when no new information or insights about HSG implementation are yielded by sampling and analysing additional papers, which will be confirmed among the research team via email); and (5) integrating the theoretical constructs into a “synthesizing argument” about HSG implementation processes (i.e. a theoretical framework) [30].

## Discussion

We will use a CIS approach to develop a theoretical framework that incorporates all facilitators, barriers and strategies for HSG implementation at four different levels (individual, organizational, community and system), that explores relationships among the above factors and strategies, and that provides an overall explanatory theory for HSG implementation. The study will have the following strengths and challenges.

### Strengths

Firstly, a CIS approach is appropriate for developing a theoretical framework based on a diverse and complex body of literature [24]. Given the characteristics of the literature on health-system policy-making and implementation, a CIS approach can be used to generate a theoretical framework for HSG implementation. Secondly, the output of our study will be the first implementation framework specifically for HSG, given the complexity of health systems, the diversity of evidence relating to health systems and the highly context-sensitive and multifactorial policy-making process, which will fill a key research gap in HSG implementation. This framework can then be widely used for different HSG with varied topics and in different contexts (including low-, middle- and high-income countries and settings). For example, policy-makers could use the framework to assess the feasibility of HSG implementation, identify facilitators that should be leveraged and barriers to be addressed, and further explore the strategies for better supporting the HSG implementation. Also, HSG developers could refer to this framework to present potential guidance about implementation facilitators, barriers and strategies when drafting the implementation section within any given HSG. Thirdly, compared with a descriptive conceptual framework, a theoretical framework for HSG implementation that includes different factors and strategies will provide clear relationships and connections among these elements. Finally, developing an HSG implementation framework will provide strong theoretical support for developing an HSG implementation tool, which will be a future step in the research programme and involve a modified Delphi method.

### Challenges and potential responses

The greatest anticipated challenge is the process of synthesizing the findings from a complex and diverse body of literature. Our response to this challenge is to enrich the CIS approach with ongoing input from our interdisciplinary research team. Also, according to our pilot search and screening, there may be few eligible documents directly relevant to HSG because the research on HSG implementation is still at a nascent stage and is just a small part of health systems research or implementation science. One response to this potential challenge is to expand the scope to include the literature not directly relevant to HSG implementation but very close or similar to our focus. For example, the indirectly relevant papers include those that are relevant to implementing CPG and/or PHG. Again, we will rely on our research team to refine the framework based on our interdisciplinary expertise and experience.

## Supplementary Information

**Additional file 1. **PRISMA-P 2015 checklist.

**Additional file 2. **Databases and search strategies.

**Additional file 3. **Data extraction form.

## Data Availability

Data sharing is not applicable to this article as no datasets were generated or analysed during the current study.

## Abbreviations

3I+E: Institutions, interests, ideas and external factors
BCW: Behaviour change wheel
BIGG: Base internacional de guias GRADE
CBM: Chinese Biomedical Literature Database
CINAHL: Cumulative Index to Nursing and Allied Health Literature
CIS: Critical interpretive synthesis
CNKI: China National Knowledge Infrastructure
CPG: Clinical practice guideline
GIN: Guidelines International Network
GRADE: Grading of Recommendations Assessment, Development and Evaluation
HSE: Health Systems Evidence
HSG: Health system guidance
NHS: National Health Service
NICE: National Institute for Health and Care Excellence
NIH: National Institutes of Health
PHG: Public health guidance
SDGs: Sustainable Development Goals
SIGN: Scottish Intercollegiate Guidelines Network
TDF: Theoretical Domains Framework
