# Supplementary material for: Facilitators, barriers and strategies for health-system guidance implementation: a critical interpretive synthesis protocol
Source: Health Res Policy Syst. 2022 Sep 29;20:105. doi: 10.1186/s12961-022-00908-0 (PMC9523963; doi:10.1186/s12961-022-00908-0)
Supplement: Supplementary file 2 — Additional file 2. Databases and search strategies. [file 12961_2022_908_MOESM2_ESM.docx]

**Additional file 2. Databases and search strategies**

| **No** | **Databases** | **Search Query** |
| --- | --- | --- |
| 1 | PubMed | 1. "Delivery of Health Care"[Mesh] 2. "health system"[Title/Abstract] 3. "health systems"[Title/Abstract] 4. "healthcare systems"[Title/Abstract] 5. "healthcare system"[Title/Abstract] 6. "health care system"[Title/Abstract] 7. "health care systems"[Title/Abstract] 8. #1-#7 OR 9. "Guidelines as Topic"[Mesh] 10. "Guideline" [Publication Type] 11. guid*[Title/Abstract] 12. recommend*[Title/Abstract] 13. statement*[Title/Abstract] 14. #9-#13 OR 15. #8 AND #14 16. "Health Planning Guidelines"[Mesh] 17. #15-#16 OR 18. "Guideline Adherence"[Mesh] 19. implement*[Title/Abstract] 20. adherence [Title/Abstract] 21. uptake [Title/Abstract] 22. utili*[Title/Abstract] 23. contextuali*[Title/Abstract] 24. #18-#23 OR 25. #17 AND #24 |
| 2 | HealthSTAR (Ovid) | 1. exp "Delivery of Health Care" 2. "health system".ab,kw,ti. 3. "health systems".ab,kw,ti. 4. "healthcare system".ab,kw,ti. 5. "healthcare systems".ab,kw,ti. 6. "health care system".ab,kw,ti. 7. "health care systems".ab,kw,ti. 8. #1-#7 OR 9. exp guideline/ 10. "guid*".ab,kw,ti. 11. "recommend*".ab,kw,ti. 12. "statement*".ab,kw,ti. 13. #9-#12 OR 14. #8 AND #13 15. exp Health Planning Guidelines/ 16. #14-#15 OR 17. exp Guideline Adherence/ 18. "implement*".ab,kw,ti. 19. adherence.ab,kw,ti. 20. uptake.ab,kw,ti. 21. "utili*".ab,kw,ti. 22. "contextuali*".ab,kw,ti. 23. #17-#22 OR 24. #16 AND #23 |
| 3 | Embase (Ovid) | 1. exp health care system/ 2. "health system".ab,kw,ti. 3. "health systems".ab,kw,ti. 4. "healthcare system".ab,kw,ti. 5. "healthcare systems".ab,kw,ti. 6. "health care system".ab,kw,ti. 7. "health care systems".ab,kw,ti. 8. #1-#7 OR 9. exp practice guideline/ 10. "guid*".ab,kw,ti. 11. "recommend*".ab,kw,ti. 12. "statement*".ab,kw,ti. 13. #9-#12 OR 14. exp protocol compliance/ 15. "implement*".ab,kw,ti. 16. "adherence".ab,kw,ti. 17. "uptake".ab,kw,ti. 18. "utili*".ab,kw,ti. 19. "contextuali*".ab,kw,ti. 20. #14-#19 OR 21. #8 AND #13 AND #20 22. limit 21 to "pubmed/medline" 23. #21 NOT #22 |
| 4 | Cochrane Library | 1. MeSH descriptor: [Delivery of Health Care] explode all trees 2. ("health system"):ti,ab,kw 3. ("health systems"):ti,ab,kw 4. ("healthcare system"):ti,ab,kw 5. ("healthcare systems"):ti,ab,kw 6. ("health care system"):ti,ab,kw 7. ("health care systems"):ti,ab,kw 8. #1-#7 OR 9. MeSH descriptor: [Guidelines as Topic] explode all trees 10. MeSH descriptor: [Guideline] explode all trees 11. MeSH descriptor: [Health Planning Guidelines] explode all trees 12. ("guid*"):ti,ab,kw 13. ("recommend*"):ti,ab,kw 14. ("statement*"):ti,ab,kw 15. #9-#14 OR 16. MeSH descriptor: [Guideline Adherence] explode all trees 17. MeSH descriptor: [Implementation Science] explode all trees 18. ("implement*"):ti,ab,kw 19. (adherence):ti,ab,kw 20. (uptake):ti,ab,kw 21. ("utili*"):ti,ab,kw 22. ("contextuali*"):ti,ab,kw 23. #16-#22 OR 24. #8 AND #15 AND #23 |
| 5 | Web of Science (Core Collection) | 1. TOPIC: ("health system") 2. TOPIC: ("health systems") 3. TOPIC: ("healthcare system") 4. TOPIC: ("healthcare systems") 5. TOPIC: ("health care system") 6. TOPIC: ("health care systems") 7. #1-#6 OR 8. TOPIC: ("guid*") 9. TOPIC: ("recommend*") 10. TOPIC: ("statement*") 11. #8-#10 OR 12. TOPIC: ("implement*") 13. TOPIC: (adherence) 14. TOPIC: (uptake) 15. TOPIC: ("utili*") 16. TOPIC: ("contextuali*") 17. #12-#16 OR 18. #7 AND #11 AND #17 |
| 6 | Cumulative Index to Nursing and Allied Health Literature (CINAHL)  (Platform: EBSCOhost) | 1. (MH "Health Care Delivery, Integrated") 2. TI "health system" OR AB "health system" 3. TI "health systems" OR AB "health systems" 4. TI "healthcare system" OR AB "healthcare system" 5. TI "healthcare systems" OR AB "healthcare systems" 6. TI "health care system" OR AB "health care system" 7. TI "health care systems" OR AB "health care systems" 8. #1-#7 OR 9. (MH "Practice Guidelines") 10. TI guid* OR AB guid* 11. TI recommend* OR AB recommend* 12. TI statement* OR AB statement* 13. #9-#12 OR 14. (MH "Guideline Adherence") 15. (MH "Implementation Science") 16. TI implement* OR AB implement* 17. TI adherence OR AB adherence 18. TI uptake OR AB uptake 19. TI utili* OR AB utili* 20. TI contextuali* OR AB contextuali* 21. #14-#20 OR 22. #8 AND #13 AND #21 23. #22 AND Limiters - Exclude MEDLINE records |
| 7 | Chinese Biomedical Literature Database (CBM) | 1. "卫生保健提供" [不加权:扩展] 2. "卫生保健提供" [常用字段:智能] 3. "卫生系统"[常用字段:智能] 4. "卫生体系"[常用字段:智能] 5. "医疗系统"[常用字段:智能] 6. "医疗体系"[常用字段:智能] 7. "医药系统"[常用字段:智能] 8. "医药体系"[常用字段:智能] 9. #1-#8 OR 10. "指南"[不加权:扩展] 11. "指南"[常用字段:智能] 12. "指引"[常用字段:智能] 13. "指导"[常用字段:智能] 14. "共识"[常用字段:智能] 15. "推荐意见"[常用字段:智能] 16. #10-#15 OR 17. "坚持准则"[不加权:扩展] 18. "坚持准则"[常用字段:智能] 19. "实施"[常用字段:智能] 20. "障碍"[常用字段:智能] 21. "促进"[常用字段:智能] 22. "策略"[常用字段:智能] 23. "阻碍"[常用字段:智能] 24. "依从"[常用字段:智能] 25. "挑战"[常用字段:智能] 26. #17-#25 OR 27. #9 AND #16 AND #26 |
| 8 | CNKI (China National Knowledge Infrastructure) | (主题:("卫生系统") + 主题:("卫生体系") + 主题:("医疗系统") + 主题:("医疗体系") + 主题:("医药系统") + 主题:("医药体系") + 主题:("卫生保健提供")) * (主题:("指南") + 主题:("指引") + 主题:("指导") + 主题:("共识") + 主题:("推荐意见")) * (主题:("实施") + 主题:("障碍") + 主题:("促进") + 主题:("策略") + 主题:("阻碍") + 主题:("依从") + 主题:("挑战") + 主题:("坚持准则"))  [+, OR; *, AND] |
| 9 | Wanfang Data | 主题:("卫生系统") + 主题:("卫生体系") + 主题:("医疗系统") + 主题:("医疗体系") + 主题:("医药系统") + 主题:("医药体系") + 主题:("卫生保健提供")) * (主题:("指南") + 主题:("指引") + 主题:("指导") + 主题:("共识") + 主题:("推荐意见")) * (主题:("实施") + 主题:("障碍") + 主题:("促进") + 主题:("策略") + 主题:("阻碍") + 主题:("依从") + 主题:("挑战") + 主题:("坚持准则")) [+, OR; *, AND] |
| 10 | Health Systems Evidence | 1. Open search: ("guid*" OR "recommend*" OR "statement*") AND ("implement*" OR adherence OR uptake OR "utili*" OR "contextuali*") 2. ("guid*" OR "recommend*" OR "statement*") AND Filter: Implementation strategies 3. #1 OR #2 |
| 11 | Google Scholar (Only browse the first 200 records) | ("health system*" OR "health care system*" OR "healthcare system*") AND ("guid*" OR "recommend*" OR "statement*") AND ("implement*" OR adherence OR uptake OR "utili*" OR "contextuali*") NOT ("clinic*" OR "treat*") |
