# Supplementary material for: Facilitators, barriers and strategies for health-system guidance implementation: a critical interpretive synthesis protocol
Source: Health Res Policy Syst. 2022 Sep 29;20:105. doi: 10.1186/s12961-022-00908-0 (PMC9523963; doi:10.1186/s12961-022-00908-0)
Supplement: Supplementary file 3 — Additional file 3. Data extraction form. [file 12961_2022_908_MOESM3_ESM.docx]

**Additional file 3. Data extraction form**

| **Data extractor** |  | | | | |
| --- | --- | --- | --- | --- | --- |
| **Document title** |  | | | | |
| **Publication year** |  | | | | |
| **Last name of first author** (if applicable, or institution) |  | | | | |
| **Publication form** | 🞎Journal article  List specific journals:  🞎Grey literature (government or organization report) | | | | |
| **Study design** (if applicable) | Primary research  🞎 Systematic review (explicit search and selection criteria)  🞎 RCT  🞎 Cross-sectional  🞎 Cohort study  🞎 Interrupted time series  🞎 Before-after study  🞎 Qualitative study  🞎 Case study  🞎 Mixed methods (select other methods as applicable)  🞎 Other (specify)  Non-research  🞎 Review (not systematic)  🞎 Theory/discussion/policy or position paper  🞎 Commentary/editorial/letter/correspondence  🞎 Website content  🞎 Guidance/guideline | | | | |
| **Country focus** (if applicable) | 🞎 High-income country(ies)  List specific countries:  🞎 Low- and middle-income country(ies)  List specific countries: | | | | |
| **Object of implementation**  (Research subject) | 🞎 HSG 🞎 CPG 🞎 PHG | | | | |
| **Funding(s)** | 🞎 Yes  List specific funding(s):  Role of funding(s):  🞎No | | | | |
| **Summary of key findings or insights from the document** (one-paragraph summary) |  | | | | |
| **Contributions to the framework (also for conceptual mapping)** | | | | | |
| **Elements/Categories** | **Individual**  (based on TDF and BCW) | **Organizational**  (based on HSE framework) | **Community**  (based on HSE framework) | **Systems** | |
|  |  |  |  | **Health system**  (based on HSE framework) | **Political system**  (based on “3I+E” framework) |
| **Facilitators** |  |  |  |  |  |
| **Barriers** |  |  |  |  |  |
| **Strategies** |  |  |  |  |  |
